# A network-biology approach for identification of key genes and pathways involved in malignant peritoneal mesothelioma

**DOI:** 10.5808/gi.21019

**Published:** 2021-06-30

**Authors:** A. M. U. B. Mahfuz, A. M. Zubair-Bin-Mahfuj, Dibya Joti Podder

**Affiliations:** 1 Department of Biotechnology & Genetic Engineering, Faculty of Life Science, University of Development Alternative, Dhaka 1209, Bangladesh; 2 Department of Oral and Maxillofacial Surgery, Dhaka Dental College, Dhaka 1216, Bangladesh; 3 Department of General Surgery, Sher-E-Bangla Medical College, Barishal 8200, Bangladesh

**Keywords:** differentially expressed genes (DEGs), hub genes, malignant peritoneal mesothelioma, miRNA, PPI network, transcription factor

## Abstract

Even in the current age of advanced medicine, the prognosis of malignant peritoneal mesothelioma (MPM) remains abysmal. Molecular mechanisms responsible for the initiation and progression of MPM are still largely not understood. Adopting an integrated bioinformatics approach, this study aims to identify the key genes and pathways responsible for MPM. Genes that are differentially expressed in MPM in comparison with the peritoneum of healthy controls have been identified by analyzing a microarray gene expression dataset. Gene Ontology and Kyoto Encyclopedia of Genes and Genomes pathway analyses of these differentially expressed genes (DEG) were conducted to gain a better insight. A protein-protein interaction (PPI) network of the proteins encoded by the DEGs was constructed using STRING and hub genes were detected analyzing this network. Next, the transcription factors and miRNAs that have possible regulatory roles on the hub genes were detected. Finally, survival analyses based on the hub genes were conducted using the GEPIA2 web server. Six hundred six genes were found to be differentially expressed in MPM; 133 are upregulated and 473 are downregulated. Analyzing the STRING generated PPI network, six dense modules and 12 hub genes were identified. Fifteen transcription factors and 10 miRNAs were identified to have the most extensive regulatory functions on the DEGs. Through bioinformatics analyses, this work provides an insight into the potential genes and pathways involved in MPM.

## Introduction

Peritoneum is the serous membrane covering the abdominal cavity and organs and is lined by a layer of simple squamous epithelium. These epithelial cells are called mesothelium [1]. Malignant peritoneal mesothelioma (MPM) is the malignancy of peritoneal mesothelial cells and is a relatively rare disease but with a very poor prognosis [2]. The 5-year relative survival rate of MPM patients is only 10% [3]. MPM is related to industrial pollutants and mineral exposure (asbestos accounts for 33%‒50% of cases, others include erionite, thorium, and mica). However, the duration between exposure and disease causation is variable. Other risk factors for MPM include familial Mediterranean fever and diffuse lymphocytic lymphoma. It usually spreads and remains within the abdominal cavity but in few cases may metastasize outside the abdomen [2,4]. MPM patients present with atypical symptoms in most cases and this is responsible for the delayed diagnosis (average time to diagnose takes 4-6 months) of this fatal disease. Abdominal symptoms include abdominal distension, ascites, abdominal pain, early satiety, intestinal obstruction, and intestinal perforation. Other non-specific symptoms include weight loss, anorexia, nausea, night sweats, and unexplained fever [2,4]. MPM may also be responsible for paraneoplastic syndrome exhibiting thrombocytosis, venous thrombosis, hypoglycemia, paraneoplastic hepatopathy, wasting of muscles, and adipose tissue [2]. Surgical intervention is the first line of treatment for MPM patients. Cytoreductive surgery with heated intraperitoneal chemotherapy is currently the most preferred treatment option. Systemic chemotherapy is chosen for those MPM patients who are unable to undergo surgery [4].

In recent years, there has been rapid advancement in microarray and RNA-sequencing (RNA-seq) technologies, and analysis of the large amount of data obtained from them has shed light on complex biological processes in an unprecedented manner. Key genes and pathways involved in different cancers and diseases have been identified by analyzing these data. These key genes can be used as biomarkers and can be utilized for early diagnosis, survival prediction, drug target identification, and drug response observation [5-11]. Bioinformatics analyses have been employed to identify important genes and pathways involved in the disease process of malignant pleural mesothelioma [12-14]. Bioinformatics approach has also identified upregulation of spliceosomal genes, especially *SF3B1* [15] and haploinsufficiency of *BAP1* gene [16] are associated with MPM. In this study, we have identified the significantly overexpressed and underexpressed genes in MPM by bioinformatics analyses.

## Methods

### Retrieval of microarray data

Gene Expression Omnibus (GEO) (https://www.ncbi.nlm.nih.gov/geo/) [17], a database of gene/microarray profiles with unrestricted public access hosted by NCBI was searched for MPM data. The following filters were applied while searching: ‘Expression profiling by array’ as the study type, ‘*Homo sapiens*’ as the organism, and the publication date to be within the last eight years. Only one MPM dataset (accession No. GSE112154) was retrieved from the search. Sciarrillo et al. [15] deposited this dataset to the GEO. They utilized Illumina HumanHT-12 V4.0 expression beadchip to obtain this gene expression profiling dataset and the dataset is based on the GPL10558 platform. Series matrix file of GSE112154 was downloaded for subsequent analyses.

### Identification of differentially expressed genes

NetworkAnalyst (https://www.networkanalyst.ca/NetworkAnalyst/faces/home.xhtml) [18], a web tool dedicated to the analysis of gene expression data was employed for analyzing the dataset of our interest. Forty-seven samples (45 malignant peritoneal mesothelioma samples and two peritoneal mesothelioma cell lines) were classified as ‘MPM’ and the rest three healthy peritoneal samples were classified as ‘Normal Mesothelium’ to make them compatible for analysis. Illumina probe IDs were converted by NetworkAnalyst to their corresponding Entrez gene IDs and official gene symbols. Probes corresponding to unannotated genes were filtered out and for multiple probes mapped to the same genes, their average expression values were considered. To obtain statistically significant results, data with the lowest 15th percentile expression and data with relative abundance lower than the 5th percentile were discarded from downstream analyses using the ‘variance’ and ‘low abundance’ filters. The dataset was quantile normalized followed by box and whisker plot visualization. After quantile normalization, data quality was assessed through three-dimensional principal component analysis (PCA). Limma (linear models for microarray data) [19], an R package for differential expression analysis of microarray data, embedded in the NetworkAnalyst server was exploited to identify the differentially expressed genes (DEGs). Genes having an adjusted p-value (Benjamini-Hochberg method) <0.05 and a |log_2_FC| value ≥1.5 were considered as differentially expressed.

### Gene Ontology and Kyoto Encyclopedia of Genes and Genomes pathway enrichment analyses:

Enrichment analyses of the DEGs were carried out using Enrichr (https://maayanlab.cloud/Enrichr/). Enrichr provides a wide range of annotations curated from other databases and annotation tools for the submitted genes [20]. A list containing official gene symbols of the DEGs was used as the input. Gene Ontology (GO) biological process, molecular function, cellular component, and Kyoto Encyclopedia of Genes and Genomes (KEGG) pathways annotations for the DEGs were retrieved through Enrichr for an insight into the roles played by these DEGs. GO [21,22] provides annotations of gene products regarding their complex biological processes, molecular functions, and cellular distributions. KEGG provides molecular-level information about large-scale biological data obtained from genome sequences and other high-throughput experiments [23]. KEGG pathway database provides maps of molecular interaction, reaction, and relation networks relevant to cellular metabolism, genetic and environmental information processing, cellular processes, organismal systems, human diseases, and drug development. GO and KEGG pathway annotations having an adjusted p-value <0.05 were considered statistically significant.

### Gene set enrichment analysis

Gene set enrichment analysis (GSEA) can be performed on DNA microarray or RNA-seq data to identify biologically linked enriched gene sets. It is a widely used approach where a priori gene sets grouped on the basis of their common biological functions, proximity in chromosomal locations, or participation in identical biological pathways are used to detect enriched gene clusters differentially expressed in two different conditions or cell types. Here the focus is placed on sets of genes rather than on individual genes [24]. The DEGs identified in our study were ranked according to their |log_2_FC| values in a .rnk file and GSEA was performed employing the GSEA Preranked module of GenePattern platform (https://www.genepattern.org/) using this file [25]. A priori annotated gene sets for conducting GSEA were retrieved from the Molecular Signatures Database (MsigDB) [26]. The c2.cp.kegg.v7.4.symbols.gmt dataset was selected as the reference gene set database. The number of gene set permutations was set to 1,000 and other parameters were used as default. In the ‘collapse dataset’ option ‘No_Collapse’ was chosen since we used official gene symbols. Gene sets having a false discovery rate (FDR) q-value <0.25 were considered significantly enriched.

### Protein-protein interaction network construction and identification of significant modules and hub genes

The network of interactions among the protein products of the DEGs was obtained utilizing STRING (Search Tool for the Retrieval of INteracting Genes) (https://string-db.org/cgi/input?sessionId=bGZ7ocKQMZ8I&input_page_active_form=multiple_sequences) [27]. The STRING database was searched with medium confidence (interaction score cutoff was 0.4) and the protein-protein interaction (PPI) network was visualized using Cytoscape 3.6.1 [28]. Applying the degree cutoff, node score cutoff, K-core, and maximum depth as 2, 0.2, 2, and 100, respectively, Molecular Complex Detection (MCODE) [29], a Cytoscape plug-in, was used to identify the modules with significant densities in the PPI network. Modules having an MCODE score ≥ 4 were considered important. CytoHubba [30], another Cytoscape plug-in was utilized for topological analysis to identify the nodes representing the hub proteins in the PPI network. CytoHubba allows to apply different methods of calculation for identifying hub nodes. 10 methods available in CytoHubba, namely ‘Betweenness,’ ‘BottleNeck,’ ‘Closeness,’ ‘Degree,’ ‘EcCentricity,’ ‘EPC’ (Edge Percolated Component), ‘MCC’ (Maximal Clique Centrality), ‘MNC’ (Maximum Neighborhood Component), ‘Radiality,’ and ‘Stress’ were applied to detect the top 50 hub nodes. Twelve proteins were ultimately identified as hub proteins from the consensus of all methods and their corresponding genes were considered as the hub genes.

### Identification of transcription factors acting on DEGs and miRNAs acting on DEGs and transcription factors

Transcription factors (TF) and microRNAs (miRNAs) are the two master regulators of gene expression. Cellular levels of TFs and miRNAs are influenced by each other in normal cells and their complex interplay controls the expression of common gene targets through feedback and feedforward loops [31]. miRNAs can bind to 3′ untranslated retion (UTR), 5′ UTR, promoter region, or even coding sequence of a gene to either suppress gene expression or induce expression. miRNAs can cause gene silencing by binding to 3′ UTR, 5′ UTR, and coding sequence, and can induce transcription by binding to the promoter region. They can also regulate gene expression within the nucleus at the time of or after transcription but the detailed mechanism of this intra-nuclear regulation by miRNAs is not yet fully understood [32]. In our study, TF-DEG, miRNA-DEG, and miRNA-TF interaction networks were identified using the miRNet web server (https://www.mirnet.ca/) [33]. miRNet is a curated database of miRNA interactions from 14 different miRNA databases. Official gene symbols of the hub genes were used as inputs and ChEA (ChIP Enrichment Analysis) was chosen as the TF database. ChEA provides data on genome-wide target specific TFs deduced from the chromatin immunoprecipitation (ChIP) followed by microarray hybridization, ChIP followed by high-throughput sequencing, ChIP with paired-end tag sequencing, and DNA adenine methyltransferase identification experiments [34].

### Identification of enriched kinases

Kinase enzymes phosphorylate proteins by transferring a phosphate group and phosphatase enzymes can dephosphorylate proteins, thus reversing the function of kinases. Their coordinated actions make possible many normal cellular processes. Dysregulated kinases and deactivated phosphatases have significant roles in different malignancies, and kinase inhibitors are promising anticancer drugs [35]. Kinases that can phosphorylate the top TFs regulating the DEGs and thereby affect their expression level were identified employing the KEA2 (Kinase Enrichment Analysis 2) webserver (https://www.maayanlab.net/KEA2/) [36]. A list of the top TFs was submitted as input to the KEA2 server. KEA2 hosts various phosphosite and protein level libraries that are either manually curated from kinase-substrate interactions in the literature or experiment-derived data. A kinase enrichment analysis can be performed against these libraries to prioritize kinases phosphorylating the query proteins. ‘Literature based kinase-substrate library’ was chosen to identify enriched kinases.

### Survival analysis of hub genes

Correlation between hub gene expression and survival rate was analyzed employing GEPIA2 (http://gepia2.cancer-pku.cn/#index) [37], a web server for gene expression analysis based on the RNA-seq data of 9,736 tumors and 8,587 normal samples from the TCGA (The Cancer Genome Atlas) and the GTEx (Genotype-Tissue Expression) projects. Since TCGA provides data for only malignant pleural mesothelioma [38] and GTEx doesn’t contain gene expression information of mesothelium, we choose the MESO (malignant pleural mesothelioma) dataset available in the GEPIA2 server to extrapolate the relationship between the expression level of hub genes and prognosis from pleural mesothelioma data. Hub gene names were used as inputs to GEPIA2. During survival analyses, median values were chosen as the group cutoff values, hazard ratios (HRs) were calculated based on Cox Proportional-Hazards Model and the analyses were conducted with a 95% confidence interval.

### Identification of protein-drug interactions

Approved drugs, investigational and experimental compounds that can interact with the identified hub proteins were identified by searching the DrugBank knowledgebase (version 5.1.7, released 2020-07-02; available at https://go.drugbank.com/) [39]. This is a rich database that currently provides information on 13,791 drug entries of which 2,653 are approved small molecule drugs, 1,417 are approved biologics, 131 are nutraceuticals and more than 6,451 are experimental drugs. Information about 5,236 non-redundant protein (i.e., drug target/enzyme/transporter/carrier) sequences related to these entries is also available through DrugBank. Official symbols of the hub proteins were used as inputs.

## Results

### Identification of differentially expressed genes

**Fig. 1.**
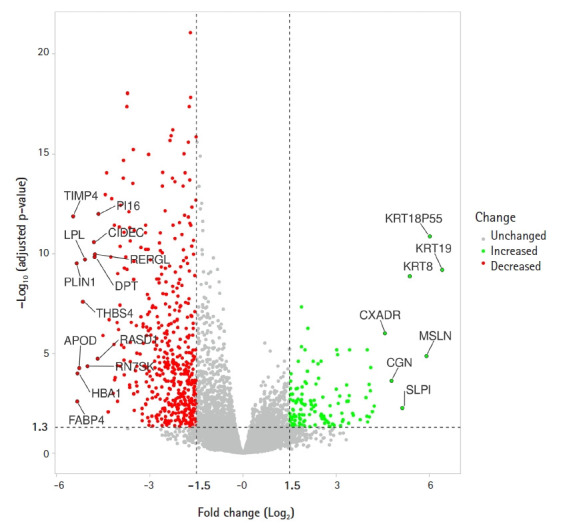
Volcano plot presentation of differentially expressed genes. The upregulated genes are shown in green dots and the downregulated genes are shown in red dots. Nonsignificant genes are shown in grey dots.

The means of the microarray samples were found uniform after quantile normalization (Supplementary Fig. 1) and the PCA plot showed MPM and normal mesothelium samples arrange in different clusters (Supplementary Fig. 2). A total number of 608 DEGs were identified from the analysis. Among these 608 DEGs, the records of two genes (Entrez gene ID: 100302207 and 100302173) have been withdrawn by the HGNC (HUGO Gene Nomenclature Committee) and these genes were excluded from subsequent analyses. Among the rest 606 DEGs, 133 genes are upregulated and 473 genes are downregulated. Fig. 1 depicts a volcano plot representing the up-and downregulated genes. This volcano plot was generated using VolcaNoseR (https://huygens.science.uva.nl/VolcaNoseR/) [40].

### GO and KEGG pathway enrichment analyses

**Fig. 2.**
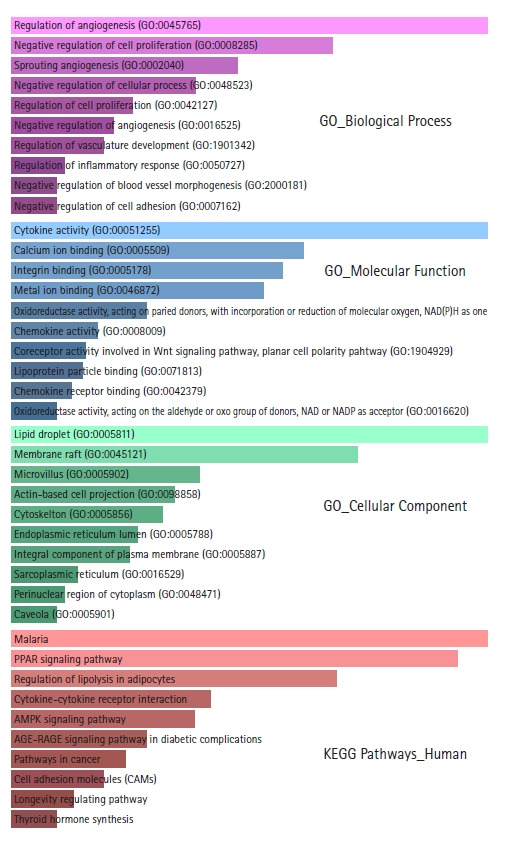
Bar graph representations of the top 10 Gene Ontology (GO) terms and enriched Kyoto Encyclopedia of Genes and Genomes (KEGG) pathways according to p-values. The pink bar graph represents GO biological processes, the sky blue bar graph represents GO molecular functions, the light green bar graph represents GO cellular components, and the light red bar graph represents enriched KEGG pathways.

GO and KEGG pathway enrichment analyses were performed using the Enrichr web server. Biological processes, molecular functions, cellular locations, and biological pathways enriched in DEGs are shown in Supplementary Tables 1‒4. From the GO biological processes, it was found that the DEGs significantly participate in the regulation of angiogenesis, negative regulation of cell proliferation, sprouting angiogenesis, negative regulation of cellular processes, regulation of cell proliferation, negative regulation of angiogenesis, regulation of vasculature development, regulation of inflammatory response, negative regulation of blood vessel morphogenesis, and in negative regulation of cell adhesion. Angiogenesis, cellular proliferation, inflammation, and cellular adhesion are critical events for tumorigenesis and metastasis. From the GO molecular functions, it was found that the DEGs mainly involve in cytokine activity, calcium ion binding, integrin binding, metal ion binding, oxidoreductase activity, chemokine activity, coreceptor activity involved in the Wnt signaling pathway, planar cell polarity pathway, lipoprotein particle binding, and chemokine receptor binding. From the GO cellular component, it was found that the proteins encoded by the DEGs are chiefly distributed in lipid droplets, membrane rafts, microvilli, actin-based cell projections, cytoskeleton, endoplasmic reticulum lumen, integral components of the plasma membrane, sarcoplasmic reticulums, perinuclear regions of cytoplasm, and caveolae. From the KEGG pathway enrichment analysis, the DEGs were found to be enriched in Malaria, PPAR signaling pathway, lipolysis regulation in adipocytes, cytokine-cytokine receptor interaction, AMPK signaling pathway, AGE-RAGE signaling pathway in diabetic complications, pathways in cancer, cell adhesion molecules, longevity regulating pathway, thyroid hormone synthesis, glycerolipid metabolism, and PI3K-Akt signaling pathway. The top 10 GO terms and enriched pathways according to p-value are depicted in Fig. 2.

### Gene set enrichment analysis

**Fig. 3.**
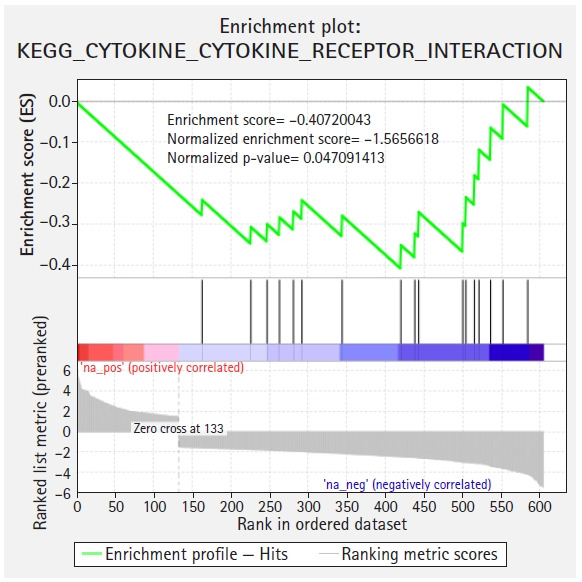
Gene set enrichment analysis identified cytokine-cytokine receptor interaction pathway is significantly disturbed in malignant peritoneal mesothelioma. KEGG, Kyoto Encyclopedia of Genes and Genomes.

GSEA was performed to corroborate the enrichment results from Enrichr. Only one pathway (cytokine-cytokine receptor interaction) was identified as downregulated from the GSEA with an FDR < 25% (FDR q = 0.0788609 and nominal p = 0.043296088) (Fig. 3). Among the 24 genes identified by Enrichr to be involved in cytokine-cytokine receptor interaction pathway (*ACVRL1*, *CCL14*, *GDF10*, *CXCL8*, *OSM*, *LIFR*, *INHBB*, *PPBP*, *NGF*, *CXCL14*, *CXCL2*, *CX3CL1*, *BMP6*, *CXCL5*, *BMP5*, *GHR*, *IL1RL1*, *IL6*, *IL18RAP*, *ACVR1C*, *CCL8*, *LEP*, *LEPR*, and *IL17D*), 17 are included in the GSEA result (*OSM*, *LEPR*, *ACVRL1*, *CXCL5*, *LIFR*, *IL18RAP*, *CCL14*, *INHBB*, *GHR*, *PPBP*, *CX3CL1*, *CXCL8*, *LEP*, *CCL8*, *CXCL2*, *CXCL14*, and *IL6*). Two underexpressed hub genes (*IL6* and *CXCL8*) are included in the GSEA outcome which further emphasizes their importance.

### PPI network construction and identification of significant modules and hub genes

**Fig. 4.**
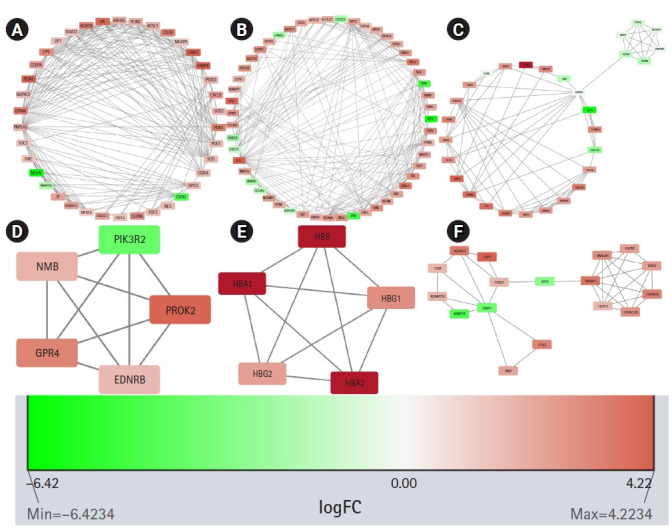
Significant modules in the protein-protein interaction (PPI) network as identified by MCODE (Molecular Complex Detection). The downregulated genes are colored red and the upregulated genes are colored green. The nodes are colored in a continuous manner according to their |log_2_FC| values. Panels A, B, C, D, E, and F are significant modules in the PPI network as identified by MCODE. The downregulated genes are colored red and the upregulated genes are colored green. The nodes are colored in a continuous manner according to their |log_2_FC| values.

STRING constructed the PPI network for the protein products of all the 606 DEGs. The number of nodes and edges in the network are 570 and 2,573, respectively. The average node degree of the network is 9.03, the average local clustering coefficient is 0.41 and the PPI enrichment p-value is <1.0e-16. The PPI network was found to have significantly more interactions than expected which is an indication of their probable biological inter-connections as a group. The PPI network was visualized in Cytoscape and the significantly dense modules were detected by the MCODE plug-in of Cytoscape. Six dense modules having an MCODE score ≥ 4 were identified. The first module has 38 nodes, 217 edges, and has an MCODE score of 11.73. The second and third modules have 51 and 29 nodes, respectively, and 224 and 79 edges, respectively. Their MCODE scores are 8.96 and 5.643, respectively. The fourth and fifth modules have five nodes, 10 edges, and their MCODE score is 5.0. The sixth module has 17 nodes, 35 edges and its MCODE score is 4.375. Proteins in each module are listed in Table 1 and are graphically presented in Fig. 4.

**Fig. 5.**
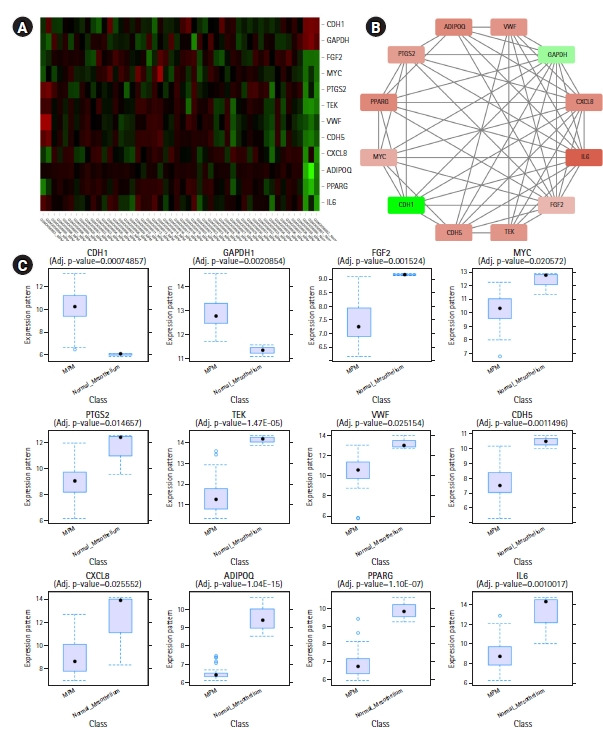
(A) Heatmap showing hub gene expressions in different samples of GSE112154. (B) Interactions among the common hub genes identified by 10 calculation methods of CytoHubba. (A, B) The downregulated genes are colored red and the upregulated genes are colored green. (B) The nodes are colored in a continuous manner according to their |log_2_FC| values. (C) Box plots showing expression levels of different hub genes in MPM (n = 47) and normal mesothelium (n = 3) samples.

CytoHubba plug-in of Cytoscape was next employed to detect the top 50 hub proteins in the network. Ten available calculation methods in CytoHubba for detecting hub proteins were used. Multiple List Comparator (http://www.molbiotools.com/listcompare.html) was utilized to identify their intersections. Twelve proteins were identified as hub proteins by all the methods and their encoding genes were considered as the high-confident key genes (Table 2). Only two of the 12 hub genes were found upregulated (*CDH1* and *GAPDH*) and the rest 10 hub genes were found downregulated. CXCL8, PTGS2, and FGF2 (fibroblast growth factor 2) were found to be present in MCODE module 1, IL6, CDH5, VWF, TEK, MYC, and CDH1 in module 2, PPARG and GAPDH in module 3, and ADIPOQ in module 6. The hub proteins were next submitted to STRING to identify their interactions which were then visualized by Cytoscape. It was found from STRING analysis that the hub proteins have significant interactions among themselves. Among the 12 hub proteins, IL6, CXCL8, FGF2, and GAPDH are each connected with the rest 11 hub proteins (Fig. 5).

### Identification of TFs acting on DEGs and miRNAs acting on DEGs and TF

**Fig. 6.**
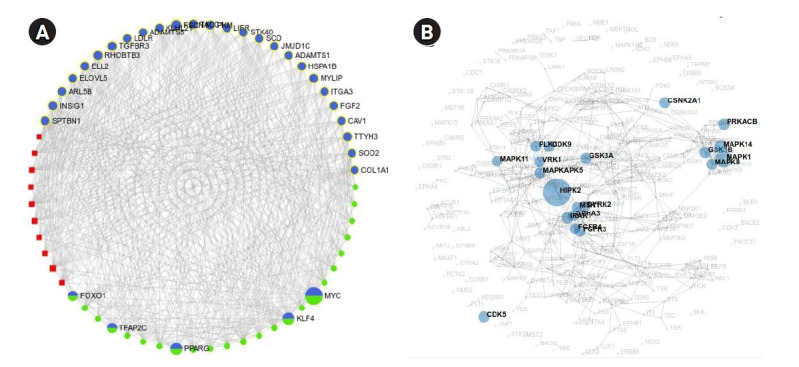
(A) Differentially expressed gene-transcription factor (TF)-miRNA interaction network. This network was obtained after filtering nodes of the original network with a degree cutoff of 150 to avoid hairball effect. In this image, genes are shown in blue circles, TFs are shown in green circles, and miRNAs are shown in red squares. (B) Network showing kinases that interact with TFs.

TFs that can act on the DEGs and miRNAs that can regulate the DEGs and TFs were identified, and the DEG-TF-miRNA interaction network was constructed and visualized through miRNet. miRNet identified 197 TFs and 2,305 miRNAs (Fig. 6A). Fifteen top TFs with a degree cutoff of 180 and 10 top miRNAs with a degree cutoff of 150 were identified. Among the 15 TFs, MYC and PPARG themselves are hub genes. Table 3 summarizes these most important TFs and miRNAs.

### Identification of enriched kinases

Enriched kinases having interactions with the identified TFs were detected through the KEA2 web server. A total number of 20 kinases having an adjusted p-value (FDR) < 0.1 were detected. Among them, significant kinases are GSK3B (6 substrates), MAPK14 (6 substrates), MAPK1 (5 substrates), CSNK2A1 (4 substrates), MAPK8 (4 substrates), HIPK2 (3 substrates), PRKACB (3 substrates), GSK3A (2 substrates), and CDK5 (2 substrates). The rest of the kinases act on single substrates. The results from KEA2 are summarized in Table 4 and are visualized in Fig. 6B.

### Survival analysis of hub genes

**Fig. 7.**
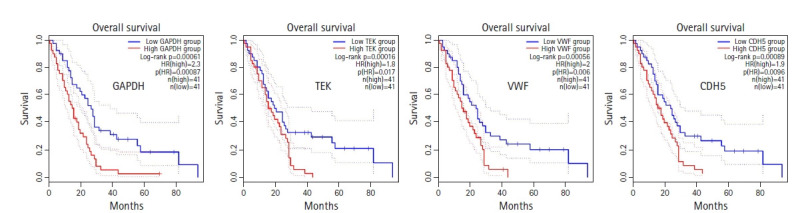
Overall survival analyses of the hub genes (p < 0.05). 95% Confidence interval is shown as dotted lines. HR, hazard ratio.

To elucidate the relationship between hub gene expression level and patient survival, survival analyses for the hub genes were performed through GEPIA2 (Fig. 7). From the survival analyses, it was found that with a log-rank p < 0.05, increased expression levels of one upregulated hub gene, *GAPDH* (HR, 2.3; p = 0.00061), and three downregulated hub genes, namely, *TEK* (HR, 1.8; p=0.016), *VWF* (HR, 2; p = 0.0056) and *CDH5* (HR, 1.9; p = 0.0089) are associated with markedly decreased overall survival duration. Moreover, Cox regression analyses of these genes indicated that these genes have high HRs (2.3, 1.8, 2, and 1.9) and can be considered as prognostic factors. These hub genes (*GAPDH*, *TEK*, *VWF*, and *CDH5*) can serve as survival biomarkers for MPM also.

### Identification of hub protein-drug interactions

The approved drugs or the compounds that can interact with the hub proteins were identified through searching DrugBank. A total number of 237 drug or drug-like compounds were found to act on the hub proteins. Seven compounds were found to act on GAPDH, eight compounds with FGF2, 2 compounds with MYC, 109 compounds with PTGS2, seven compounds with TEK, 11 compounds with VWF, two compounds with CDH5, four compounds with CXCL8, 74 compounds with PPARG, and 13 compounds with IL6. No drug/compound was found to act on CDH1 and ADIPOQ. Among the identified compounds, there are agonists, antagonists, and compounds with still unknown pharmacological actions. For the two upregulated hub proteins (CDH1 and GAPDH), no antagonist/inhibitor was found. A full list of the identified compounds is available in Supplementary Table 5 and the hub protein-drug interaction network can be found in Supplementary Fig. 3.

## Discussion

MPM is an aggressive disease and its prognosis is usually very poor. Identification of biomarkers of this disease can help in early diagnosis and treatment, and observing responses to ongoing treatment. For this purpose, in this study, we have analyzed GSE112154, a microarray dataset containing gene expression information of normal peritoneum and MPM. We have found a total number of 606 genes are differentially expressed in MPM (133 genes are upregulated and 473 genes are downregulated) with an adjusted p < 0.05 and a |log_2_FC| value ≥ 1.5. These DEGs were next subjected to GO and KEGG pathway enrichment analyses followed by GSEA. From the GSEA, it was found that many genes involved in the cytokine-cytokine receptor interaction pathway are significantly downregulated in MPM, thereby interfering with the normal functions of this pathway.

Six significant modules and 12 hub genes (*CDH1*, *GAPDH*, *FGF2*, *MYC*, *PTGS2*, *TEK*, *VWF*, *CDH5*, *CXCL8*, *ADIPOQ*, *PPARG*, and *IL6*) were identified from our analyses. Two of these hub genes, *CDH1* and *GAPDH*, are overexpressed and the rest 10 hub genes are underexpressed. CDH1 or E-cadherin’s main role is in cell-cell adhesion. *CDH1* is considered a tumor suppressor gene [41] but was found overexpressed in epithelioid malignant pleural mesothelioma [42]. GAPDH (glyceraldehyde-3-phosphate dehydrogenase) is a glycolysis enzyme that can be found in all tissues. Glyceraldehyde-3-phosphate is converted to 1,3-diphosphoglycerate in the presence of GAPDH. In addition to this catalytic conversion, GAPDH also takes part in various other complex biological processes like replication and repair of DNA, export of tRNA from the nucleus, exo- and endocytosis, cytoskeletal organization, etc. *GAPDH* overexpression is associated with different types of lung cancer, renal cell carcinoma, glioma, breast cancer, hepatocellular carcinoma, pancreatic cancer, gastric and colorectal cancer, melanoma, prostate, and urinary bladder cancer. In some of these cancers, *GAPDH* overexpression was responsible for chemotherapeutic resistance [43]. *GAPDH* is also overexpressed in malignant pleural mesothelioma [44]. From survival analysis of pleural mesothelioma, it can be extrapolated that *GAPDH* upregulation would also be associated with reduced survival in peritoneal mesothelioma. FGF2 protein has important roles in cellular proliferation, motility, and differentiation. *FGF2* gene has a suppressive effect on *CDH1*. Overexpression of the *FGF2* gene in ovarian cancer cells was associated with downregulation of *CDH1*, upregulation of *Slug* (*SNAI2*) and *ZEB1*, and increased invasiveness [45]. We have found an inverse relationship between *CDH1* and *FGF2* in MPM. We have found *CDH1* is significantly upregulated and *FGF2* is downregulated in MPM. We have also found *SNAI2* is downregulated and *ZEB1* is not differentially expressed in MPM. This suggests a vice versa regulatory relationship may exist between *CDH1* and *FGF2* in MPM. Increased FGF2 protein level also causes a receptor‐independent upregulation of the *IL6* gene [46]. This implies a directly proportional relationship between *FGF2* and *IL6* exists. We have found both *FGF2* and *IL6* are underexpressed in MPM. *MYC* is a proto-oncogene and MYC protein has roles in cellular proliferation, differentiation, apoptosis, cellular senescence, DNA damage responses, biosynthesis of ribosome, glycolysis, and mitochondrial functions. It can initiate events that lead to either hyperproliferation of cancer cells or prevention of tumorigenesis. It was further observed that tumor cells having poor blood supply becomes metabolically inactive and MYC level is decreased in these cells. MYC helps survive these cells under hypoglycemic and hypoxic conditions. *MYC* is usually overexpressed in different cancers. However, it has been reported that *MYC* is underexpressed in adrenocortical cancers. [47-49]. We have also found it is underexpressed in MPM. *PTGS2* (also known as *COX-2*) is overexpressed in many solid tumors, for example, breast, colorectal, lung, pancreatic, liver, and ovarian cancers [50]. But we have found this gene is downregulated in MPM. TEK is a receptor tyrosine kinase protein. It is involved in angiogenesis through TEK-angiopoietin 1 (ANGPT1) and TEK-angiopoietin 2 (ANGPT2) signaling. ANGPT1 is an agonist of TEK whereas ANGPT2 can play as both an agonist and an antagonist. *TEK* is downregulated in metastatic clear cell renal cell carcinoma and is associated with poor prognosis [51]. We have found both *TEK* and *ANGPT1* are downregulated in MPM. Zhang et al. (2020) [12] showed that *CXCL8/IL8*, *PPARG*, *ADIPOQ*, and *IL6* are upregulated in malignant pleural mesothelioma. But we have found from our analyses that these genes are downregulated in MPM. These genes might have different roles in the pathogenesis of malignant pleural mesothelioma and MPM. We have found *VWF* and *CDH5* are downregulated in MPM. These two genes are also downregulated in non-small cell lung cancer [52]. Apart from the hub genes, we have found the most upregulated genes in MPM are *KRT19*, *KRT18P55*, *MSLN*, *KRT8*, *SLPI*, *CGN*, and *CXADR* (Coxsackie and adenovirus receptor). *KRT19* (keratin 19) and *KRT8* (keratin 8) are upregulated in lung adenocarcinoma where they are associated with poor prognosis. High *KRT19* expression is also associated with liver and breast cancer. High *KRT8* expression was also observed in clear cell renal cell carcinoma and gastric cancer [53]. *KRT8* was also found to be upregulated in rat models of mesothelioma [54]. *KRT18P55* (keratin 18 pseudogene 55) encodes a long intergenic noncoding RNA (KRT18P55), is overexpressed in intestinal-type gastric cancer, and correlates with its progression [55]. This implies that this long noncoding RNA has a role common in intestinal-type gastric cancer and MPM. *MSLN* (mesothelin) in healthy individuals is expressed in pleura, pericardium and peritoneum. However, it is upregulated in all types of mesothelioma, pancreatic adenocarcinoma, ovarian cancers, lung adenocarcinoma, and cholangiocarcinoma [56,57]. *SLPI* (secretory leukocyte protease inhibitor) upregulation has been observed in breast, lung, stomach, and colorectal cancers [58]. CXADR is a receptor for Coxsackie B viruses and adenoviruses 2 and 5 [59]. It was shown that CXADR maintains survival and growth of oral squamous cell carcinoma by translocating CDH1 from cytoplasm to cell membrane [60]. We have found that both *CXADR* and *CDH1* are upregulated in MPM and postulate a similar role played by *CXADR* in MPM.

TFs and miRNAs maintain spatiotemporal gene expression. TFs and miRNAs that can regulate the DEGs were also identified. Among the identified miRNAs, mir-34a was reported to be downregulated in MPM in comparison with normal peritoneum. Re-expression of mir-34a in MPM cells exhibited oncosuppressive events both *in vitro* and *in vivo*. This suggests downregulation of this miRNA has a possible role in the pathogenesis of MPM [61]. The roles of other miRNAs are yet to be elucidated.

In this study, adopting a biological network analysis approach, we have identified the potential pathways and genes involved in MPM. These candidate genes and pathways need to be validated in further *in vitro* and *in vivo* experiments and in MPM samples to confirm their active roles and to manipulate them for clinical usefulness.

## Figures and Tables

**Table 1.** Significant modules in the PPI having an MCODE score ≥ 4

Module	Nodes	Edges	MCODE score	Protein
1	38	217	11.73	TF, GPC3, PDK4, MSLN, ACACB, CLDN5, NES, FGF2, CIDEC, SDC2, CDH2, CEBPA, AGPAT2, CXCL8, CXCL5, PNPLA2, CX3CL1, CIDEA, SOD2, LPL, GPT, PLIN1, SPARCL1, PLIN2, HRC, GPAM, SCD, FABP4, LIPE, TMEM132A, PTGS2, DGAT2, ACSL1, ABHD5, CD36, MLXIPL, MFGE8, PCK1
2	51	224	8.96	VTN, MYC, SLC2A5, SELP, MCAM, HBEGF, NAMPT, CDH5, PTPRB, HMOX1, CAV1, KRT8, MCEMP1, TEK, MRAP, SNAI2, CDC20, SOX17, PDE3B, STOM, ALDH3B1, UBE2C, CAT, KLHL21, LPAR5, S1PR3, CXCL2, MYLIP, FBXO2, ZBTB16, SPSB1, VAMP8, S1PR1, FBXO7, HECW2, PPBP, NGF, LDLR, AGTR1, PECAM1, VWF, ADCY4, ANGPT1, CDH1, RBP4, MMP25, IL6, STAT5A, CFD, SELE, LEPR
3	29	79	5.643	THRSP, SOD3, KLF4, GPX3, GPX8, ADRB2, PTGER4, NOX4, CA9, THBD, RAMP2, INSIG1, NOS3, ASPM, NUSAP1, PPARG, TOP2A, KRT19, CEP55, NEK2, PARPBP, CALCRL, VIPR1, S100A4, GAPDH, PTH1R, COL1A1, GPBAR1, LEP
4	5	10	5	PIK3R2, NMB, PROK2, EDNRB, GPR4
5	5	10	5	HBA2, HBB, HBA1, HBG2, HBG1
6	17	35	4.375	ADAMTS5, HSPH1, DNAJB1, HSPB7, ATF4, FOXO1, BAG3, PTX3, TIMP1, ANG, MMP24, HSPA1B, ADIPOQ, HSPA12B, DNAJA4, OSM, EBF1

PPI, protein-protein interaction; MOCDE, Molecular Complex Detection.

**Table 2.** Twelve hub genes with their respective log_2_FC and adjusted p-values (FDR)

Hub gene	Log_2_FC	Adj. p-value (FDR)
*CDH1*	4.1017	0.00074857
*GAPDH*	1.5141	0.0020854
*FGF2*	‒1.7636	0.001524
*MYC*	‒2.0953	0.020572
*PTGS2*	‒2.4941	0.014657
*TEK*	‒2.7234	1.47E-05
*VWF*	‒2.7452	0.025154
*CDH5*	‒2.7455	0.0011496
*CXCL8*	‒3.0163	0.025552
*ADIPOQ*	‒3.0367	1.04E-15
*PPARG*	‒3.0666	1.10E-07
*IL6*	‒4.1746	0.0010017

FDR, false discovery rate.

**Table 3.** Top transcription factors and miRNAs

Transcription factor	Degree	Betweenness	miRNA	Degree	Betweenness
SOX2	293	14,615.15	hsa-mir-124-3p	241	75,109.76
MYC	283	20,679.36	hsa-mir-16-5p	236	75,393.02
SUZ12	229	7,521.775	hsa-mir-1-3p	209	57,945.4
EGR1	226	8,460.82	hsa-mir-27a-3p	195	49,269.11
STAT3	211	7,224.139	hsa-mir-129-2-3p	184	36,612.51
HNF4A	211	7,456.627	hsa-mir-34a-5p	170	36,162.74
NANOG	209	6,106.242	hsa-mir-155-5p	167	39,779.95
SPI1	208	8,373.637	hsa-mir-146a-5p	166	32,701.75
TP63	207	7,455.71	hsa-mir-374a-5p	159	30,642.76
AR	206	8,356.316	hsa-let-7b-5p	155	40,806.08
PPARG	204	9,683.092			
RUNX1	193	6,815.653			
TP53	187	7,209.77			
MITF	185	5,649.56			
POU5F1	181	4,757.409			

miRNA, microRNA.

**Table 4.** Kinases acting on the TFs regulating the hub genes

Kinase name	Adj. p-value (FDR)	No. of substrate TFs	Substrates (TF)
HIPK2	0.0003872	3	RUNX1, TP53, STAT3
MAPK1	0.0146	5	PPARG, TP53, STAT3, MYC, AR
MAPK14	0.0445	6	PPARG, RUNX1, HNF4A, STAT3, TP53, MYC
GSK3A	0.0722	2	TP53, MYC
CSNK2A1	0.0745	4	EGR1, TP53, SPI1, MYC
MAPK8	0.0745	4	TP53, MYC, PPARG, STAT3
GSK3B	0.0745	6	MITF, RUNX1, TP53, PPARG, STAT3, MYC
EPHA3	0.0745	1	STAT3
DYRK2	0.0745	1	STAT3
IRAK1	0.0745	1	STAT3
PRKACB	0.0745	3	EGR1, TP53, MITF
CDK5	0.0745	2	STAT3, TP53
PLK3	0.0784	1	TP53
CDK9	0.0784	1	TP53
VRK1	0.0806	1	TP53
FGFR4	0.0806	1	STAT3
MAPKAPK5	0.0806	1	TP53
MAPK11	0.0863	1	HNF4A
MSK1	0.0863	1	STAT3
FGFR3	0.0909	1	STAT3

TF, transcription factor; FDR, false discovery rate.

## Supplementary Materials

Supplemental Table 1. Highly significant biological processes (adjusted p < 0.01) participated by differentially expressed genes

Supplemental Table 2. Highly significant molecular functions (adjusted p < 0.1) performed by differentially expressed genes

Supplemental Table 3. Most probable cellular locations (adjusted p-value <0.05) of the proteins encoded by differentially expressed genes

Supplemental Table 4. Highly significant (adjusted p-value <0.05) biological pathways enriched in differentially expressed genes

Supplemental Table 5. Drugs/chemical compounds that can interact with the hub proteins

Supplemental Fig. 1. Box plot presentation of microarray samples. The left-sided box plots represent data before normalization and the right-sided box plots represent data after normalization.

Supplemental Fig. 2. Three-dimensional principal component analysis (PCA) plot shows clustering of normal mesothelium and malignant peritoneal mesothelioma (MPM) samples present in the GSE112154 dataset.

Supplemental Fig. 3. Hub protein-Drug Interaction network (visualized in Cytoscape). The hub proteins are colored green and the drug compounds are colored cyan.
