# Supplementary material for: A network-biology approach for identification of key genes and pathways involved in malignant peritoneal mesothelioma
Source: Genomics Inform. 2021 Jun 30;19(2):e16. doi: 10.5808/gi.21019 (PMC8261271; doi:10.5808/gi.21019)
Supplement: Supplemental Table 1. — Highly significant biological processes (adjusted p < 0.01) participated by differentially expressed genes [file gi-21019suppl1.pdf]

Supplementary Table 1. Highly significant biological processes (adjusted p < 0.01) participated by differentially expressed genes

GO biological process	Adjusted p-value
Regulation of angiogenesis (GO:0045765)	7.599E-07
Negative regulation of cell proliferation (GO:0008285)	1.541E-05
Sprouting angiogenesis (GO:0002040)	9.334E-05
Negative regulation of cellular process (GO:0048523)	0.00019
Regulation of cell proliferation (GO:0042127)	0.00063
Negative regulation of angiogenesis (GO:0016525)	0.00082
Regulation of vasculature development (GO:1901342)	0.00089
Regulation of inflammatory response (GO:0050727)	0.00192
Negative regulation of blood vessel morphogenesis (GO:2000181)	0.00204
Negative regulation of cell adhesion (GO:0007162)	0.00184
Positive regulation of intracellular signal transduction (GO:1902533)	0.00193
Regulation of endothelial cell proliferation (GO:0001936)	0.00180
Regulation of cardiac conduction (GO:1903779)	0.00192
Positive regulation of cell proliferation (GO:0008284)	0.00223
Regulation of anatomical structure morphogenesis (GO:0022603)	0.00215
Cell-cell junction organization (GO:0045216)	0.00320
Positive regulation of sprouting angiogenesis (GO:1903672)	0.00457
Regulation of sequestering of triglyceride (GO:0010889)	0.00469
Negative regulation of cell migration (GO:0030336)	0.00479
Negative regulation of signal transduction (GO:0009968)	0.00471
Regulation of macrophage derived foam cell differentiation (GO:0010743)	0.00475
Positive regulation of protein phosphorylation (GO:0001934)	0.00572
Cell-cell junction assembly (GO:0007043)	0.00550
Cellular response to low-density lipoprotein particle stimulus (GO:0071404)	0.00557
Positive regulation of glutamate receptor signaling pathway (GO:1900451)	0.00551
Oxygen transport (GO:0015671)	0.00530
Valine metabolic process (GO:0006573)	0.00510
Positive regulation of cellular biosynthetic process (GO:0031328)	0.00492
Neutrophil mediated immunity (GO:0002446)	0.00650
Vasculogenesis (GO:0001570)	0.00663
Cell-cell adhesion mediated by cadherin (GO:0044331)	0.00800
Response to insulin (GO:0032868)	0.00833
Cellular response to cytokine stimulus (GO:0071345)	0.00971
Cell junction assembly (GO:0034329)	0.00977
Positive regulation of angiogenesis (GO:0045766)	0.00956
Positive regulation of inflammatory response (GO:0050729)	0.00944
Neutrophil activation involved in immune response (GO:0002283)	0.00985
