# Supplementary material for: A network-biology approach for identification of key genes and pathways involved in malignant peritoneal mesothelioma
Source: Genomics Inform. 2021 Jun 30;19(2):e16. doi: 10.5808/gi.21019 (PMC8261271; doi:10.5808/gi.21019)
Supplement: Supplemental Table 2. — Highly significant molecular functions (adjusted p < 0.1) performed by differentially expressed genes [file gi-21019suppl2.pdf]

Supplementary Table 2. Highly significant molecular functions (adjusted p < 0.1) performed by differentially expressed genes

GO molecular function	Adjusted p-value
Cytokine activity (GO:0005125)	0.00128
Calcium ion binding (GO:0005509)	0.01124
Integrin binding (GO:0005178)	0.01005
Metal ion binding (GO:0046872)	0.01021
Oxidoreductase activity, acting on paired donors, with incorporation or reduction of molecular Oxygen, NAD(P)H as one donor, and incorporation of one atom of oxygen (GO:0016709)	0.04736
Chemokine activity (GO:0008009)	0.08246
Coreceptor activity involved in Wnt signaling pathway, planar cell polarity pathway (GO:1904929)	0.08460
Lipoprotein particle binding (GO:0071813)	0.07671
Chemokine receptor binding (GO:0042379)	0.08159
Oxidoreductase activity, acting on the aldehyde or oxo group of donors, NAD or NADP as Acceptor (GO:0016620)	0.09217

Supplementary Table 3. Most probable cellular locations (adjusted p-value <0.05) of the proteins encoded by differentially expressed genes

GO cellular location	Adjusted p-value
lipid droplet (GO:0005811)	0.00018
membrane raft (GO:0045121)	0.00069
microvillus (GO:0005902)	0.00473
actin-based cell projection (GO:0098858)	0.00514
cytoskeleton (GO:0005856)	0.00494
endoplasmic reticulum lumen (GO:0005788)	0.00592
integral component of plasma membrane (GO:0005887)	0.00572
sarcoplasmic reticulum (GO:0016529)	0.01093
perinuclear region of cytoplasm (GO:0048471)	0.01152
caveola (GO:0005901)	0.01179
sarcoplasm (GO:0016528)	0.0117
cytoplasmic vesicle lumen (GO:0060205)	0.02049
cortical cytoskeleton (GO:0030863)	0.03153
ficolin-1-rich granule lumen (GO:1904813)	0.04082
catenin complex (GO:0016342)	0.04087
platelet alpha granule (GO:0031091)	0.04458
actin cytoskeleton (GO:0015629)	0.04316
specific granule membrane (GO:0035579)	0.04282
