# Supplementary material for: A network-biology approach for identification of key genes and pathways involved in malignant peritoneal mesothelioma
Source: Genomics Inform. 2021 Jun 30;19(2):e16. doi: 10.5808/gi.21019 (PMC8261271; doi:10.5808/gi.21019)
Supplement: Supplemental Table 4. — Highly significant (adjusted p-value <0.05) biological pathways enriched in differentially expressed genes [file gi-21019suppl4.pdf]

Supplementary Table 4. Highly significant (adjusted p-value <0.05) biological pathways enriched in differentially expressed genes

**KEGG pathway**	**Adjusted p-value**
Malaria	5.38E-06
PPAR signaling pathway	6.10E-06
Regulation of lipolysis in adipocytes	6.50E-05
Cytokine-cytokine receptor interaction	0.00087
AMPK signaling pathway	0.001
AGE-RAGE signaling pathway in diabetic complications	0.00251
Pathways in cancer	0.00351
Cell adhesion molecules (CAMs)	0.0051
Longevity regulating pathway	0.00895
Thyroid hormone synthesis	0.0118
Glycerolipid metabolism	0.01344
PI3K-Akt signaling pathway	0.01341
Non-alcoholic fatty liver disease (NAFLD)	0.01443
Complement and coagulation cascades	0.01374
Aldosterone synthesis and secretion	0.01571
ECM-receptor interaction	0.0158
HIF-1 signaling pathway	0.01623
Fluid shear stress and atherosclerosis	0.01831
Adipocytokine signaling pathway	0.01789
JAK-STAT signaling pathway	0.0204
Phenylalanine metabolism	0.02114
Insulin resistance	0.02264
Human papillomavirus infection	0.03576
Apelin signaling pathway	0.03874
Proteoglycans in cancer	0.04084
Cortisol synthesis and secretion	0.04044
Glucagon signaling pathway	0.04594
Tyrosine metabolism	0.04756
Transcriptional misregulation in cancer	0.04709
Proximal tubule bicarbonate reclamation	0.04768
cGMP-PKG signaling pathway	0.04672
Parathyroid hormone synthesis, secretion and action	0.04685
African trypanosomiasis	0.04551
