# Supplementary material for: A network-biology approach for identification of key genes and pathways involved in malignant peritoneal mesothelioma
Source: Genomics Inform. 2021 Jun 30;19(2):e16. doi: 10.5808/gi.21019 (PMC8261271; doi:10.5808/gi.21019)
Supplement: Supplemental Table 5. — Drugs/chemical compounds that can interact with the hub proteins [file gi-21019suppl5.pdf]

Supplementary Table 5. Drugs/chemical compounds that can interact with the hub proteins

DrugBank ID	Name	Drug Group	Pharmaco-logical Actions	Role
**CDH1**				
No relevant drug found				
**GAPDH**				
DB00157	NADH	Approved, nutraceutical	Unknown	
DB02059	Adenosine-5-Diphosphoribose	Experimental	Unknown	
DB03893	Thionicotinamide-Adenine-Dinucleotide	Experimental	Unknown	
DB07347	4-(2-Aminoethyl)Benzenesulfonyl Fluoride	Experimental	Unknown	
DB09130	Copper	Approved, investigational	Unknown	
DB09092	Xanthinol	Approved, withdrawn	Yes	Cofactor
DB11638	Artenimol	Experimental, investigational	Unknown	Ligand
**FGF2**				
DB00686	Pentosan polysulfate	Approved	Yes	Antagonist
DB03935	1,4-Dideoxy-O2-Sulfo-Glucuronic Acid	Experimental	Unknown	
DB03959	N,O6-Disulfo-Glucosamine	Experimental	Unknown	
DB03981	1,4-Dideoxy-5-Dehydro-O2-Sulfo-Glucuronic Acid	Experimental	Unknown	
DB05434	ABT-510	Investigational	Unknown	
DB00877	Sirolimus	Approved, investigational	Yes	Other/ Unknown
DB00364	Sucralfate	Approved	Yes	Agonist/ Inducer
DB01109	Heparin	Approved, investigational	Unknown	
**MYC Proto-Oncogene Protein**				
DB08813	Nadroparin	Approved, investigational	Unknown	Inhibitor
DB00945	Acetylsalicylic acid	Approved, vet_approved	Unknown	Downregulator
**PTGS2**				
DB00154	Dihomo-gamma-linolenic acid	Investigational, nutraceutical	Yes	
DB00159	Icosapent	Approved, nutraceutical	Yes	Inhibitor
DB00480	Lenalidomide	Approved	Unknown	Negative Modulator
DB00482	Celecoxib	Approved, investigational	Yes	Inhibitor
DB00533	Rofecoxib	Approved, investigational, withdrawn	Yes	Inhibitor
DB00580	Valdecoxib	Approved, investigational,	Yes	Inhibitor

		withdrawn		
DB00605	Sulindac	Approved, investigational	Yes	Inhibitor
DB00712	Flurbiprofen	approved, investigational	Yes	Inhibitor
DB00784	Mefenamic acid	Approved	Yes	Inhibitor
DB00812	Phenylbutazone	Approved, vet_approved	Yes	Inhibitor
DB00821	Carprofen	Approved, vet_approved, withdrawn	Yes	Inhibitor
DB00991	Oxaprozin	Approved	Yes	Inhibitor
DB00244	Mesalazine	Approved	Yes	Inhibitor
DB00316	Acetaminophen	Approved	Yes	Inhibitor
DB00328	Indomethacin	Approved, investigational	Yes	Inhibitor
DB00461	Nabumetone	Approved	Yes	Inhibitor
DB00465	Ketorolac	Approved	Yes	Inhibitor
DB00469	Tenoxicam	Approved	Yes	Inhibitor
DB00500	Tolmetin	Approved	Yes	Inhibitor
DB00573	Fenoprofen	Approved	Yes	Inhibitor
DB00586	Diclofenac	Approved, vet_approved	Yes	Inhibitor
DB00749	Etodolac	Approved, investigational, vet_approved	Yes	Inhibitor
DB00788	Naproxen	Approved, vet_approved	Yes	Inhibitor
DB00814	Meloxicam	Approved, vet_approved	Yes	Inhibitor
DB00861	Diflunisal	Approved, investigational	Yes	Inhibitor
DB00870	Suprofen	Approved, withdrawn	Yes	Inhibitor
DB00939	Meclofenamic acid	Approved, vet_approved	Yes	Inhibitor
DB00945	Acetylsalicylic acid	Approved, vet_approved	Yes	Inhibitor
DB00963	Bromfenac	Approved	Yes	Inhibitor
DB01009	Ketoprofen	Approved, vet_approved	Yes	Inhibitor
DB01014	Balsalazide	Approved, investigational	Yes	Inhibitor
DB01050	Ibuprofen	Approved	Yes	Inhibitor
DB01283	Lumiracoxib	Approved, investigational	Yes	Inhibitor
DB01399	Salsalate	Approved	Yes	Inhibitor
DB00936	Salicylic acid	Approved, investigational, vet_approved	Yes	Inhibitor
DB01041	Thalidomide	Approved, investigational,	Unknown	Antagonist

		withdrawn		
DB01404	Ginseng	Approved, investigational, nutraceutical	Unknown	Inhibitor
DB01628	Etoricoxib	Approved, investigational	Yes	Inhibitor
DB01600	Tiaprofenic acid	Approved	Yes	Inhibitor
DB04743	Nimesulide	Approved, investigational, withdrawn	Yes	Inhibitor
DB02709	Resveratrol	Investigational	Unknown	Inhibitor
DB04725	Licofelone	Investigational	Unknown	Inhibitor
DB05095	Cimicoxib	Investigational	Unknown	Inhibitor
DB02266	Flufenamic acid	Approved	Unknown	Inhibitor
DB00554	Piroxicam	Approved, investigational	Yes	Inhibitor
DB01397	Magnesium salicylate	Experimental	Yes	Inhibitor
DB00250	Dapsone	Approved, investigational	Unknown	Substrate
DB01041	Thalidomide	Approved, investigational, withdrawn	Unknown	Substrate
DB06725	Lornoxicam	Approved, investigational	Yes	Inhibitor
DB03866	Prostaglandin G2	Experimental	Unknown	
DB03477	1-Phenylsulfonamide-3-Trifluoromethyl-5-Parabromophenylpyrazole	Experimental	Unknown	
DB06802	Nepafenac	Approved, investigational	Unknown	Inhibitor
DB00795	Sulfasalazine	Approved	Yes	Inhibitor
DB04552	Niflumic acid	Experimental	Yes	Inhibitor
DB01435	Antipyrine	Approved, investigational	Unknown	Inhibitor
DB01419	Antrafenine	Approved	Unknown	Inhibitor
DB01401	Choline magnesium trisalicylate	Approved	Unknown	Inhibitor
DB00233	Aminosalicylic acid	Approved	Unknown	Inhibitor
DB00963	Bromfenac	Approved	Unknown	Inhibitor
DB00887	Bumetanide	Approved	Unknown	Inhibitor
DB06774	Capsaicin	Approved	Unknown	Inhibitor
DB00856	Chlorphenesin	Approved, experimental	Unknown	Inhibitor
DB05095	Cimicoxib	Investigational	Unknown	Inhibitor
DB00720	Clodronic acid	Approved, investigational, vet_approved	Unknown	Inhibitor
DB06195	Seliciclib	Investigational	Unknown	Inhibitor
DB00035	Desmopressin	Approved	Unknown	Inducer
DB05804	Prasterone sulfate	Investigational	Unknown	Inducer
DB01395	Drospirenone	Approved	Unknown	Inhibitor/ Inducer
DB06804	Nonoxynol-9	Approved, withdrawn	Unknown	Inducer

DB00515	Cisplatin	Approved	Unknown	Inhibitor
DB00884	Risedronic acid	Approved, investigational	Unknown	Inducer
DB00360	Sapropterin	Approved, investigational	Unknown	Inducer
DB06436	Semaxanib	Investigational	Unknown	Inducer
DB05875	Sar9, Met (O2)11-Substance P	Investigational	Unknown	Inducer
DB00041	Aldesleukin	Approved	Unknown	Inducer
DB00620	Triamcinolone	Approved, vet_approved	Unknown	Inhibitor
DB08819	Tafluprost	Approved	Unknown	Inducer
DB08910	Pomalidomide	Approved	Yes	Inhibitor
DB00773	Etoposide	Approved	Unknown	Substrate
DB08439	Parecoxib	Approved	Yes	Inhibitor
DB09217	Firocoxib	Experimental, vet_approved	Unknown	
DB13167	Alclofenac	Approved, withdrawn	Yes	Antagonist
DB09213	Dexibuprofen	Approved, investigational	Yes	Inhibitor
DB11133	Omega-3 fatty acids	Approved, nutraceutical	Unknown	Substrate
DB13168	Omega-6 fatty acids	Nutraceutical	Unknown	Substrate
DB06736	Aceclofenac	Approved, investigational	Yes	Inhibitor
DB13783	Acemetacin	Approved, investigational	Yes	Antagonist
DB09215	Droxicam	Withdrawn	Yes	Inhibitor
DB09212	Loxoprofen	Approved	Yes	Antagonist
DB09216	Tolfenamic acid	Approved, investigational	Yes	Antagonist
DB09214	Dexketoprofen	Approved, investigational	Unknown	Antagonist
DB09214	Dexketoprofen	Approved, investigational	Unknown	Inhibitor
DB09295	Talniflumate	Experimental	Unknown	Antagonist
DB09295	Talniflumate	Experimental	Unknown	Inhibitor
DB09285	Morniflumate	Experimental	Unknown	
DB09285	Morniflumate	Experimental	Unknown	Inhibitor
DB09288	Propacetamol	Experimental	Yes	Antagonist
DB11079	Trolamine salicylate	Approved	Yes	Inhibitor
DB11071	Phenyl salicylate	Approved	Unknown	Antagonist
DB13346	Bufexamac	Approved, withdrawn	Yes	Inhibitor
DB11327	Dipyrithione	Approved	Unknown	
DB13961	Fish oil	Approved, nutraceutical	Yes	Inhibitor
DB11323	Glycol salicylate	Approved	Yes	Antagonist
DB11201	Menthyl salicylate	Approved	Yes	Antagonist
DB13501	Bendazac	Approved, withdrawn	Unknown	
DB09061	Cannabidiol	Approved,	Unknown	Inhibitor

		investigational		
DB14011	Nabiximols	Investigational	Unknown	Inhibitor
DB14009	Medical Cannabis	Experimental, investigational	Unknown	Inhibitor
DB11752	Bryostatin 1	Investigational	Unknown	Inducer
**TEK**				
DB08221	N-{4-METHYL-3-[(3-PYRIMIDIN-4-YLPYRIDIN-2-YL)AMINO]PHENYL}-3-(TRIFLUOROMETHYL)BENZAMIDE	Experimental	Unknown	
DB05294	Vandetanib	Approved	Unknown	Inhibitor
DB08896	Regorafenib	Approved	Yes	Inhibitor
DB08901	Ponatinib	Approved, investigational	Unknown	Inhibitor
DB00415	Ampicillin	Approved, vet_approved	Unknown	
DB12010	Fostamatinib	Approved, investigational	Unknown	Inhibitor
DB14840	Ripretinib	Approved	Yes	Inhibitor
**VWF**				
DB00025	Antihemophilic factor, human recombinant	Approved, investigational	Yes	Binder
DB05202	Egaptivon pegol	investigational	Unknown	
DB06081	Caplacizumab	Approved, investigational	Unknown	
DB09108	Simoctocog alfa	Approved	Yes	Binding
DB11606	Susoctocog alfa	Approved, investigational	Yes	Binding
DB11607	Efmoroctocog alfa	Approved, investigational	Yes	Binding
DB09329	Antihemophilic Factor (Recombinant), PEGylated	Approved, investigational	Yes	Binder
DB09329	Antihemophilic Factor (Recombinant), PEGylated	Approved, investigational	Unknown	
DB13998	Lonoctocog alfa	Approved, investigational	Yes	Binder
DB13999	Moroctocog alfa	Approved	Yes	Binder
DB14738	Turoctocog alfa pegol	Approved	Yes	Binder
**CDH5**				
DB05685	FX06	Investigational	Unknown	
DB00480	Lenalidomide	Approved	Unknown	Antagonist
**CXCL8/IL8**				
DB05434	ABT-510	Investigational	Unknown	
DB05484	MDX-018	Investigational	Unknown	
DB05855	Rivanicline	Investigational	Unknown	Antagonist
DB06083	Tapinarof	Investigational	Unknown	
**ADIPOQ**				
No Relevant Drug Found				
**PPARG**				
DB00197	Troglitazone	Approved, investigational, withdrawn	Yes	Agonist
DB00412	Rosiglitazone	Approved,	Yes	Agonist

		investigational		
DB01132	Pioglitazone	Approved, investigational	Yes	Agonist
DB00159	Icosapent	Approved, nutraceutical	Yes	Agonist
DB04971	Reglitazar	Investigational	Unknown	
DB04689	2-{5-[3-(6-BENZOYL-1-PROPYLNAPHTHALEN-2-YLOXY)PROPOXY]INDOL-1-YL}ETHANOIC ACID	Experimental	Unknown	
DB05187	Elafibranor	investigational	Unknown	
DB05490	AMG-131	investigational	Yes	Partial agonist
DB05854	CLX-0921	investigational	Unknown	
DB11811	Arhalofenate	investigational	Unknown	
DB12662	Naveglitazar	investigational	Unknown	
DB06510	Muraglitazar	investigational	Unknown	
DB06521	Ertiprotafib	investigational	Unknown	
DB06533	Ragaglitazar	investigational	Unknown	
DB06536	Tesaglitazar	investigational	Unknown	
DB00328	Indomethacin	Approved, investigational	Unknown	Activator
DB00731	Nateglinide	Approved, investigational	Unknown	Agonist
DB00912	Repaglinide	Approved, investigational	Unknown	Agonist
DB01252	Mitiglinide	Approved, investigational	Unknown	Agonist
DB01067	Glipizide	Approved, investigational	Unknown	Agonist
DB00966	Telmisartan	Approved, investigational	Yes	Partial agonist
DB01014	Balsalazide	Approved, investigational	Yes	Agonist
DB01393	Bezafibrate	Approved, investigational	Yes	Agonist
DB06908	(2S)-3-(1-{[2-(2-CHLOROPHENYL)-5-METHYL-1,3-OXAZOL-4-YL]METHYL}-1H-INDOL-5-YL)-2-ETHOXYPROPANOIC ACID	Experimental	Unknown	
DB06926	(9Z,11E,13S)-13-hydroxyoctadeca-9,11-dienoic acid	Experimental	Unknown	
DB07053	2-{5-[3-(7-PROPYL-3-TRIFLUOROMETHYLBENZO[D]ISOXAZOL-6-YLOXY)PROPOXY]INDOL-1-YL}ETHANOIC ACID	Experimental	Unknown	
DB07111	(4S,5E,7Z,10Z,13Z,16Z,19Z)-4-hydroxydocosa-5,7,10,13,16,19-hexaenoic acid	Experimental	Unknown	
DB07172	(5R,6E,8Z,11Z,14Z,17Z)-5-hydroxyicosa-6,8,11,14,17-pentaenoic acid	Experimental	Unknown	
DB07208	(8E,10S,12Z)-10-hydroxy-6-oxooctadeca-8,12-dienoic acid	Experimental	Unknown	
DB07209	(8R,9Z,12Z)-8-hydroxy-6-oxooctadeca-9,12-	Experimental	Unknown	

	dienoic acid			
DB07302	9(S)-HODE	Experimental	Unknown	
DB07509	difluoro(5-{2-[(5-octyl-1H-pyrrol-2-yl-kappaN)methylidene]-2H-pyrrol-5-yl-kappaN}pentanoato)boron	Experimental	Unknown	
DB07675	(2S)-2-ETHOXY-3-{4-[2-(10H-PHENOXAZIN-10-YL)ETHOXY]PHENYL}PROPANOIC ACID	Experimental	Unknown	
DB07723	3-(5-methoxy-1H-indol-3-yl)propanoic acid	Experimental	Unknown	
DB07724	Indeglitazar	Experimental	Unknown	
DB07842	(2S)-2-(4-ethylphenoxy)-3-phenylpropanoic acid	Experimental	Unknown	
DB07863	2-chloro-5-nitro-N-phenylbenzamide	Experimental	Unknown	
DB08121	(2S)-2-(biphenyl-4-yloxy)-3-phenylpropanoic acid	Experimental	Unknown	
DB08302	3-[5-(2-nitropent-1-en-1-yl)furan-2-yl]benzoic acid	Experimental	Unknown	
DB08402	2-[(2,4-DICHLOROBENZOYL)AMINO]-5-(PYRIMIDIN-2-YLOXY)BENZOIC ACID	Experimental	Unknown	
DB08435	(5E,14E)-11-oxoprosta-5,9,12,14-tetraen-1-oic acid	Experimental	Unknown	
DB08560	3-FLUORO-N-[1-(4-FLUOROPHENYL)-3-(2-THIENYL)-1H-PYRAZOL-5-YL]BENZENESULFONAMIDE	Experimental	Unknown	
DB04270	(S)-3-(4-(2-Carbazol-9-Yl-Ethoxy)-Phenyl)-2-Ethoxy-Propionic Acid	Experimental	Unknown	
DB08760	(2S)-2-(4-chlorophenoxy)-3-phenylpropanoic acid	Experimental	Unknown	
DB00244	Mesalazine	Approved	Yes	Agonist
DB00795	Sulfasalazine	Approved	Yes	Agonist
DB01050	Ibuprofen	Approved	Unknown	Activator
DB08915	Aleglitazar	Investigational	Yes	Agonist
DB09213	Dexibuprofen	Approved, investigational	Unknown	Activator
DB11133	Omega-3 fatty acids	Approved, nutraceutical	Yes	Ligand
DB03756	Doconexent	Approved, investigational	Yes	Ligand
DB09198	Lobeglitazone	Experimental	Yes	Activator
DB09006	Clinofibrate	Experimental	Unknown	
DB09201	Ciglitazone	Experimental	Unknown	
DB11672	Curcumin	Approved, experimental, investigational	Unknown	
DB00132	alpha-Linolenic acid	Approved, investigational, nutraceutical	Unknown	
DB02746	Phthalic Acid	Experimental	Unknown	
DB02709	Resveratrol	Investigational	Unknown	
DB01118	Amiodarone	Approved, investigational	Unknown	Agonist
DB00313	Valproic acid	Approved, investigational	Unknown	

DB03600	Capric acid	Experimental	Unknown	Ligand
DB04224	Oleic Acid	Approved, investigational, vet_approved	Unknown	Ligand
DB08604	Triclosan	Approved, investigational	Unknown	
DB00573	Fenoprofen	Approved	Unknown	
DB02266	Flufenamic acid	Approved	Unknown	Agonist
DB09061	Cannabidiol	Approved, investigational	Unknown	Activator
DB13873	Fenofibric acid	Approved	Unknown	
DB14011	Nabiximols	Investigational	Unknown	
DB14009	Medical Cannabis	Experimental, investigational	Unknown	
DB13961	Fish oil	Approved, nutraceutical	Unknown	
DB14034	Darglitazone	Experimental	Unknown	Agonist
DB12007	Isoflavone	Experimental	Unknown	Agonist
DB14635	Curcumin sulfate	Experimental	Unknown	
DB00845	Clofazimine	Approved, investigational	Unknown	Modulator
**IL6**				
DB01404	Ginseng	Approved, investigational, nutraceutical	Unknown	Antagonist
DB05017	YSIL6	Investigational	Unknown	
DB05470	VX-702	Investigational	Unknown	
DB05513	Atiprimod	Investigational	Unknown	
DB05744	CRx-139	Investigational	Unknown	
DB05767	Andrographolide	Investigational	Unknown	
DB06083	Tapinarof	Investigational	Unknown	
DB09036	Siltuximab	Approved, investigational	Yes	Antagonist/ Antibody
DB09221	Polaprezinc	Experimental	Unknown	Inhibitor
DB10770	Foreskin fibroblast (neonatal)	Approved	Unknown	Agonist
DB10772	Foreskin keratinocyte (neonatal)	Approved	Yes	Agonist
DB11967	Binimetinib	Approved, investigational	Unknown	
DB12140	Dilmapimod	Investigational	Unknown	
