# Supplementary material for: A network-biology approach for identification of key genes and pathways involved in malignant peritoneal mesothelioma
Source: Genomics Inform. 2021 Jun 30;19(2):e16. doi: 10.5808/gi.21019 (PMC8261271; doi:10.5808/gi.21019)
Supplement: Supplemental Fig. 1. — Box plot presentation of microarray samples. The left-sided box plots represent data before normalization and the right-sided box plots represent data after normalization. [file gi-21019suppl6.pdf]

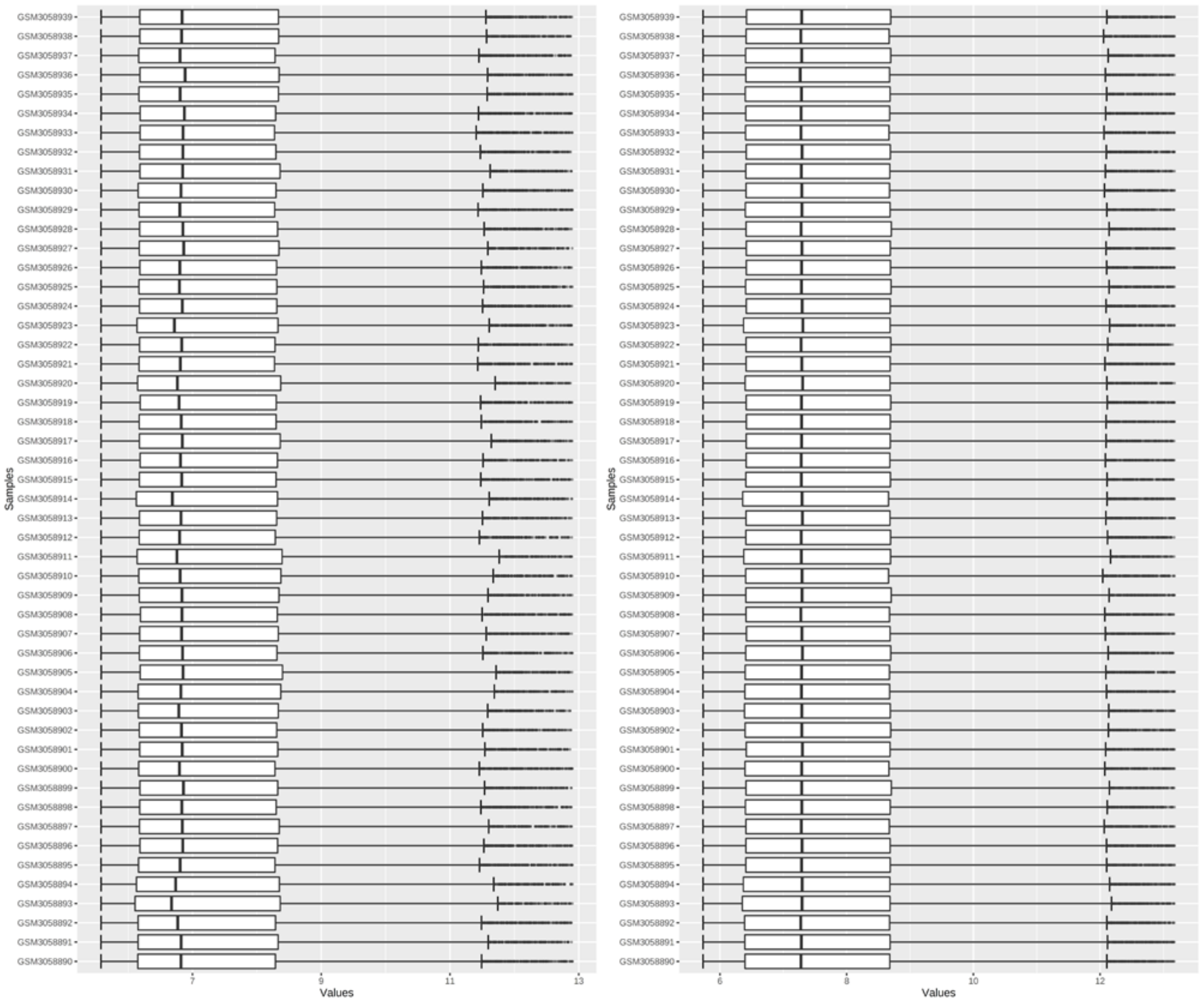

Supplementary Fig. 1. Box plot presentation of microarray samples. The left-sided box plots represent data before normalization and the right-sided box plots represent data after normalization.
