# Supplementary material for: A network-biology approach for identification of key genes and pathways involved in malignant peritoneal mesothelioma
Source: Genomics Inform. 2021 Jun 30;19(2):e16. doi: 10.5808/gi.21019 (PMC8261271; doi:10.5808/gi.21019)
Supplement: Supplemental Fig. 2. — Three-dimensional principal component analysis (PCA) plot shows clustering of normal mesothelium and malignant peritoneal mesothelioma (MPM) samples present in the GSE112154 dataset. [file gi-21019suppl7.pdf]

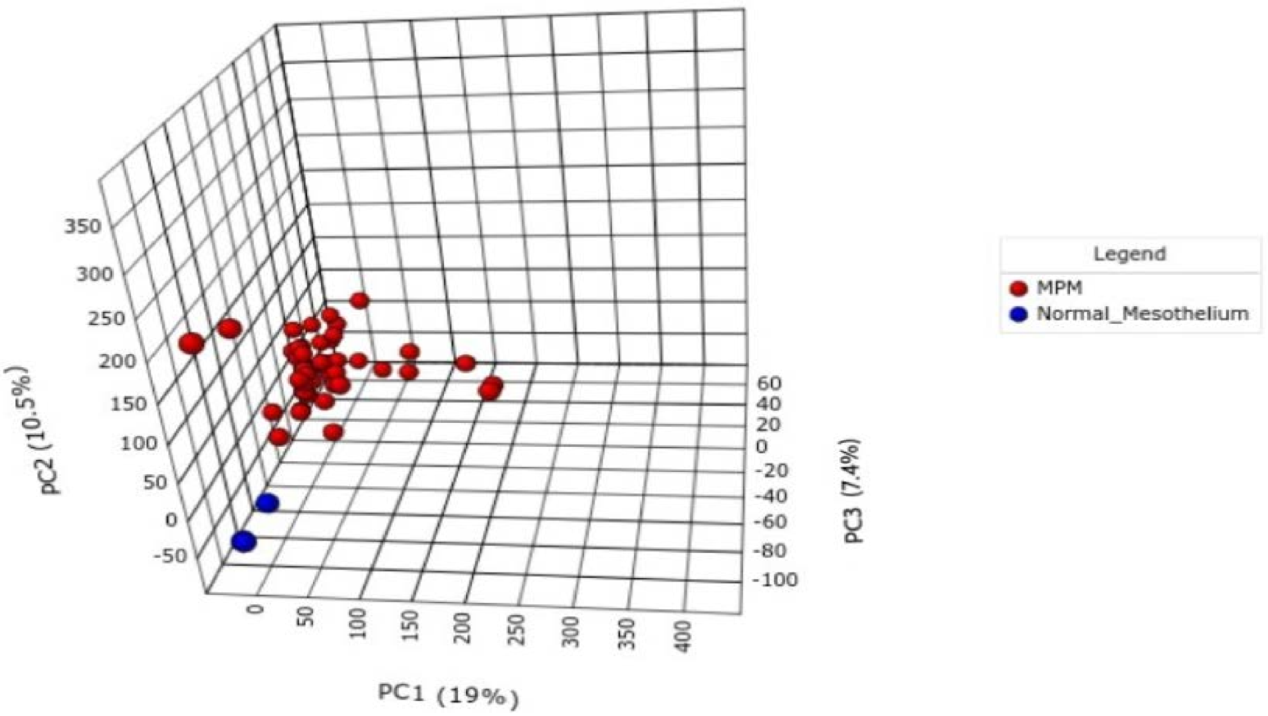

Supplementary Fig. 2. Three-dimensional principal component analysis (PCA) plot shows clustering of normal mesothelium and malignant peritoneal mesothelioma (MPM) samples present in the GSE112154 dataset.
