# Supplementary material for: A network-biology approach for identification of key genes and pathways involved in malignant peritoneal mesothelioma
Source: Genomics Inform. 2021 Jun 30;19(2):e16. doi: 10.5808/gi.21019 (PMC8261271; doi:10.5808/gi.21019)
Supplement: Supplemental Fig. 3. — Hub protein-Drug Interaction network (visualized in Cytoscape). The hub proteins are colored green and the drug compounds are colored cyan. [file gi-21019suppl8.pdf]

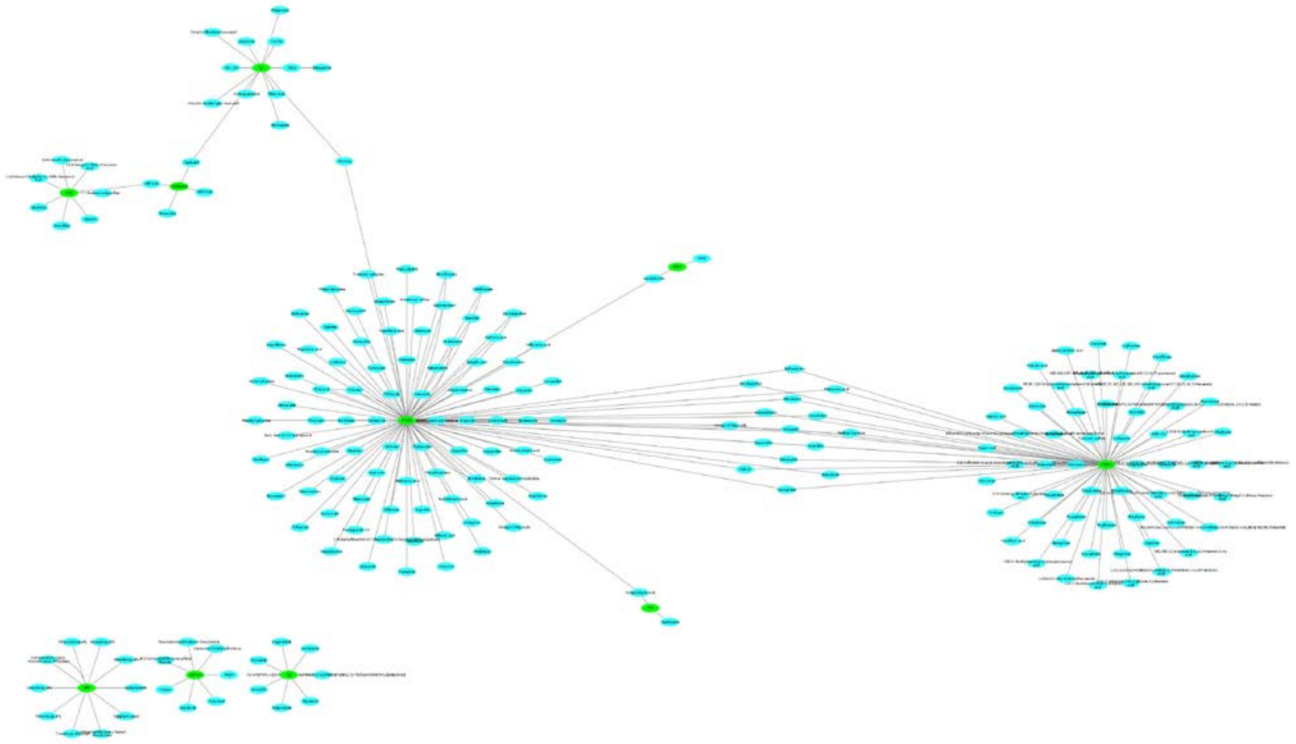

**Supplementary Fig. 3.** Hub protein-Drug Interaction network (visualized in Cytoscape). The hub proteins are colored green and the drug compounds are colored cyan.
